# 2-Methyl­sulfanyl-1,2,4-triazolo[1,5-*a*]quinazolin-5(4*H*)-one

**DOI:** 10.1107/S1600536812021757

**Published:** 2012-05-19

**Authors:** Rashad Al-Salahi, Mohamed Al-Omar, Mohammed Abbas, Seik Weng Ng

**Affiliations:** a Department of Pharmaceutical Chemistry, College of Pharmacy, King Saud University, Riyadh 11451, Saudi Arabia; b Department of Chemistry, University of Malaya, 50603 Kuala Lumpur, Malaysia; c Chemistry Department, Faculty of Science, King Abdulaziz University, PO Box 80203 Jeddah, Saudi Arabia

## Abstract

The non-H atoms of the title compound, C_10_H_8_N_4_OS, lie approximately in a common plane (r.m.s. deviation = 0.058 Å). In the crystal, two mol­ecules are linked across a center of inversion by a pair of N—H⋯N hydrogen bonds, forming a a dimer.

## Related literature

For the synthesis, see: Al-Salahi & Geffken (2011 ▶). For a related compound, see: Al-Salahi *et al.* (2011 ▶).
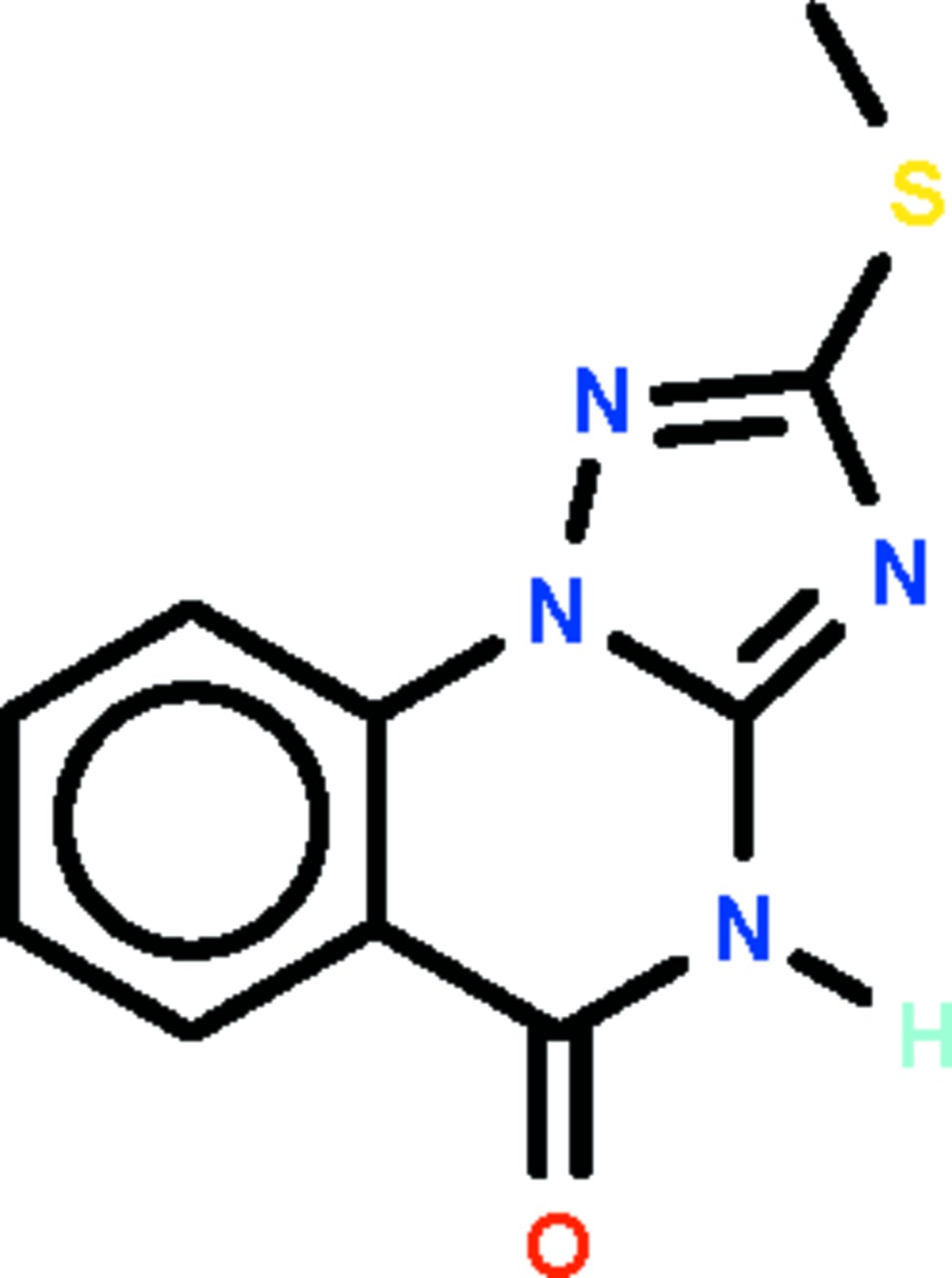


## Experimental

### 

#### Crystal data

C_10_H_8_N_4_OS
*M* *_r_* = 232.26
Monoclinic, 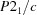

*a* = 10.4150 (1) Å
*b* = 5.0631 (1) Å
*c* = 18.6564 (3) Å
β = 96.857 (1)°
*V* = 976.76 (3) Å^3^
*Z* = 4
Cu *K*α radiation
μ = 2.81 mm^−1^
*T* = 294 K
0.35 × 0.15 × 0.10 mm

#### Data collection

Agilent SuperNova Dual diffractometer with an Atlas detector
Absorption correction: multi-scan (*CrysAlis PRO*; Agilent, 2012 ▶) *T*_min_ = 0.439, *T*_max_ = 0.766
15980 measured reflections
2052 independent reflections
1996 reflections with *I* > 2σ(*I*)
*R*_int_ = 0.033

#### Refinement

*R*[*F* ^2^ > 2σ(*F* ^2^)] = 0.030
*wR*(*F* ^2^) = 0.089
*S* = 1.05
2052 reflections
151 parameters
H atoms treated by a mixture of independent and constrained refinement
Δρ_max_ = 0.24 e Å^−3^
Δρ_min_ = −0.21 e Å^−3^

### 

Data collection: *CrysAlis PRO* (Agilent, 2012 ▶); cell refinement: *CrysAlis PRO*; data reduction: *CrysAlis PRO*; program(s) used to solve structure: *SHELXS97* (Sheldrick, 2008 ▶); program(s) used to refine structure: *SHELXL97* (Sheldrick, 2008 ▶); molecular graphics: *X-SEED* (Barbour, 2001 ▶); software used to prepare material for publication: *publCIF* (Westrip, 2010 ▶).

## Supplementary Material

Crystal structure: contains datablock(s) global, I. DOI: 10.1107/S1600536812021757/bt5914sup1.cif

Structure factors: contains datablock(s) I. DOI: 10.1107/S1600536812021757/bt5914Isup2.hkl

Supplementary material file. DOI: 10.1107/S1600536812021757/bt5914Isup3.cml

Additional supplementary materials:  crystallographic information; 3D view; checkCIF report

## Figures and Tables

**Table 1:** Hydrogen-bond geometry (Å, °)

*D*—H⋯*A*	*D*—H	H⋯*A*	*D*⋯*A*	*D*—H⋯*A*
N4—H4⋯N3^i^	0.85 (2)	2.05 (2)	2.896 (2)	174 (2)

Symmetry code: (i) 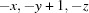.

**Fig. 1.**
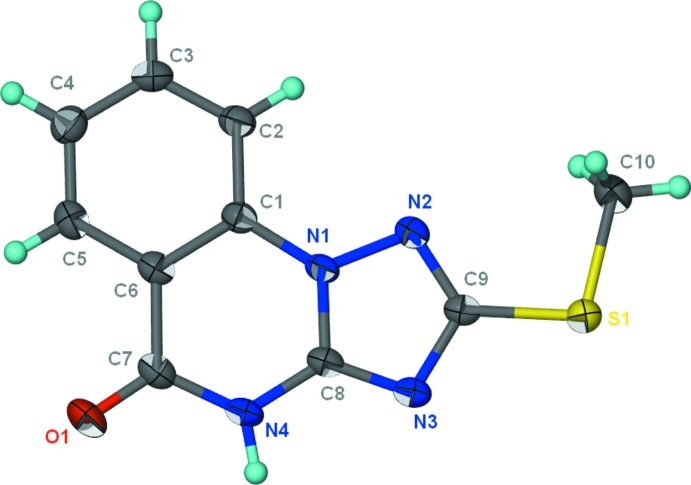
Anisotropic displacement ellipsoid plot (Barbour, 2001) of C10H8N4OS at the 70% probability level; hydrogen atoms are drawn as spheres of arbitrary radius.

## supplementary crystallographic information

### Comment

The title compound (Scheme I) was synthesized from 2-hydrazinobenzoic acid and dimethyl *N*-cyanoimidodithiocarbonate; further reactions on the inherent lactam unit yielded other derivatives (Al-Salahi & Geffken, 2011). The non-H atoms of C_10_H_8_N_4_OS lie in a common plane (Fig. 1). Two molecules are linked across a center-of-inversion by N–H···N hydrogen bonds to form a dimer (Table 1). A related compound that has a benzyloxy group instead of the methylsulfanyl group also exists as a hydrogen-bonded dimer (Al-Salahi *et al.*, 2011).

### Experimental

Under ice-cold conditions, 2-hydrazinobenzoic acid (10 mmol, 1.52 g) was added to a solution of dimethyl *N*-cyanodithioimidocarbonate (10 mmol, 1.46 g) in ethanol (20 ml). Triethylamine (30 mmol, 3.03 g) was added. The reaction mixture was stirred overnight at room temperature. Concentrated hydrochloric acid was added; the acidified mixture for heated for an hour. The mixture was poured into ice water; the solid that formed was collected and recrystallized from ethanol to give colorless crystals.

### Refinement

All H-atom were located in a difference Fourier map. Carbon-bound H-atoms were placed in calculated positions [C–H 0.93 to 0.96 Å, *U*_iso_(H) 1.2 to 1.5*U*_eq_(C)] and were included in the refinement in the riding model approximation.

The amino H-atom was freely refined.

### Crystal data

**Table:**

C_10_H_8_N_4_OS	*F*(000) = 480
*M**_r_* = 232.26	*D*_x_ = 1.579 Mg m^−^^3^
Monoclinic, *P*2_1_/*c*	Cu *K*α radiation, λ = 1.54184 Å
Hall symbol: -P 2ybc	Cell parameters from 10679 reflections
*a* = 10.4150 (1) Å	θ = 4.3–76.7°
*b* = 5.0631 (1) Å	µ = 2.81 mm^−^^1^
*c* = 18.6564 (3) Å	*T* = 294 K
β = 96.857 (1)°	Prism, colorless
*V* = 976.76 (3) Å^3^	0.35 × 0.15 × 0.10 mm
*Z* = 4	

### Data collection

**Table:**

Agilent SuperNova Dual diffractometer with an Atlas detector	2052 independent reflections
Radiation source: SuperNova (Cu) X-ray Source	1996 reflections with *I* > 2σ(*I*)
Mirror monochromator	*R*_int_ = 0.033
Detector resolution: 10.4041 pixels mm^-1^	θ_max_ = 76.9°, θ_min_ = 4.3°
ω scan	*h* = −13→13
Absorption correction: multi-scan (*CrysAlis PRO*; Agilent, 2012)	*k* = −6→6
*T*_min_ = 0.439, *T*_max_ = 0.766	*l* = −23→23
15980 measured reflections	

### Refinement

**Table:**

Refinement on *F*^2^	Secondary atom site location: difference Fourier map
Least-squares matrix: full	Hydrogen site location: inferred from neighbouring sites
*R*[*F*^2^ > 2σ(*F*^2^)] = 0.030	H atoms treated by a mixture of independent and constrained refinement
*wR*(*F*^2^) = 0.089	*w* = 1/[σ^2^(*F*_o_^2^) + (0.0547*P*)^2^ + 0.2828*P*] where *P* = (*F*_o_^2^ + 2*F*_c_^2^)/3
*S* = 1.05	(Δ/σ)_max_ = 0.001
2052 reflections	Δρ_max_ = 0.24 e Å^−^^3^
151 parameters	Δρ_min_ = −0.21 e Å^−^^3^
0 restraints	Extinction correction: *SHELXL97* (Sheldrick, 2008), Fc^*^=kFc[1+0.001xFc^2^λ^3^/sin(2θ)]^-1/4^
Primary atom site location: structure-invariant direct methods	Extinction coefficient: 0.0089 (9)

### Fractional atomic coordinates and isotropic or equivalent isotropic displacement parameters (Å^2^)

**Table:**

	*x*	*y*	*z*	*U*_iso_*/*U*_eq_	
S1	0.07695 (3)	0.09449 (7)	0.203762 (17)	0.03356 (15)	
O1	0.19589 (12)	1.1102 (2)	−0.05498 (6)	0.0527 (3)	
N1	0.25468 (10)	0.6522 (2)	0.12100 (5)	0.0284 (2)	
N2	0.24857 (10)	0.4808 (2)	0.17833 (5)	0.0299 (2)	
N3	0.07817 (11)	0.4211 (2)	0.09036 (6)	0.0327 (3)	
N4	0.13609 (11)	0.7645 (3)	0.01027 (6)	0.0365 (3)	
C1	0.34851 (12)	0.8433 (3)	0.11483 (6)	0.0282 (3)	
C2	0.45835 (13)	0.8685 (3)	0.16496 (7)	0.0331 (3)	
H2	0.4716	0.7559	0.2045	0.040*	
C3	0.54687 (13)	1.0631 (3)	0.15483 (8)	0.0357 (3)	
H3	0.6209	1.0803	0.1877	0.043*	
C4	0.52720 (14)	1.2342 (3)	0.09627 (8)	0.0381 (3)	
H4A	0.5867	1.3674	0.0908	0.046*	
C5	0.41916 (13)	1.2058 (3)	0.04634 (7)	0.0365 (3)	
H5	0.4066	1.3195	0.0070	0.044*	
C6	0.32892 (12)	1.0088 (3)	0.05434 (7)	0.0311 (3)	
C7	0.21660 (14)	0.9725 (3)	−0.00156 (7)	0.0359 (3)	
C8	0.15228 (12)	0.6134 (3)	0.07104 (7)	0.0303 (3)	
C9	0.14125 (12)	0.3490 (3)	0.15648 (7)	0.0291 (3)	
C10	0.18994 (15)	0.0881 (3)	0.28453 (8)	0.0443 (4)	
H10A	0.1713	−0.0598	0.3138	0.066*	
H10B	0.1826	0.2487	0.3111	0.066*	
H10C	0.2763	0.0721	0.2718	0.066*	
H4	0.072 (2)	0.722 (4)	−0.0199 (11)	0.062 (6)*	

### Atomic displacement parameters (Å^2^)

**Table:**

	*U*^11^	*U*^22^	*U*^33^	*U*^12^	*U*^13^	*U*^23^
S1	0.0327 (2)	0.0351 (2)	0.0319 (2)	−0.00273 (12)	−0.00017 (14)	0.00156 (12)
O1	0.0552 (7)	0.0637 (8)	0.0356 (6)	−0.0047 (5)	−0.0093 (5)	0.0204 (5)
N1	0.0297 (5)	0.0316 (5)	0.0225 (5)	0.0011 (4)	−0.0028 (4)	0.0021 (4)
N2	0.0315 (5)	0.0323 (6)	0.0249 (5)	0.0010 (4)	−0.0010 (4)	0.0025 (4)
N3	0.0308 (5)	0.0381 (6)	0.0273 (5)	−0.0004 (4)	−0.0038 (4)	0.0003 (4)
N4	0.0364 (6)	0.0439 (7)	0.0263 (5)	−0.0022 (5)	−0.0087 (4)	0.0045 (5)
C1	0.0298 (6)	0.0300 (6)	0.0246 (6)	0.0029 (5)	0.0018 (5)	−0.0023 (5)
C2	0.0345 (6)	0.0356 (7)	0.0275 (6)	0.0012 (5)	−0.0031 (5)	0.0007 (5)
C3	0.0328 (6)	0.0399 (7)	0.0328 (7)	−0.0007 (5)	−0.0020 (5)	−0.0048 (5)
C4	0.0394 (7)	0.0364 (7)	0.0387 (7)	−0.0050 (6)	0.0051 (6)	−0.0020 (6)
C5	0.0428 (7)	0.0356 (7)	0.0312 (7)	0.0012 (6)	0.0049 (6)	0.0046 (5)
C6	0.0335 (6)	0.0335 (7)	0.0258 (6)	0.0039 (5)	0.0015 (5)	0.0000 (5)
C7	0.0385 (7)	0.0413 (7)	0.0269 (6)	0.0034 (6)	−0.0008 (5)	0.0035 (6)
C8	0.0310 (6)	0.0340 (7)	0.0245 (6)	0.0030 (5)	−0.0029 (5)	−0.0009 (5)
C9	0.0291 (6)	0.0314 (6)	0.0260 (6)	0.0027 (5)	0.0000 (5)	−0.0023 (5)
C10	0.0442 (8)	0.0510 (9)	0.0354 (7)	−0.0051 (6)	−0.0041 (6)	0.0098 (6)

### Geometric parameters (Å, º)

**Table:**

S1—C9	1.7402 (14)	C1—C6	1.4008 (18)
S1—C10	1.7980 (15)	C2—C3	1.378 (2)
O1—C7	1.2145 (18)	C2—H2	0.9300
N1—C8	1.3443 (15)	C3—C4	1.390 (2)
N1—N2	1.3848 (15)	C3—H3	0.9300
N1—C1	1.3897 (17)	C4—C5	1.3800 (19)
N2—C9	1.3237 (17)	C4—H4A	0.9300
N3—C8	1.3192 (18)	C5—C6	1.391 (2)
N3—C9	1.3759 (16)	C5—H5	0.9300
N4—C8	1.3614 (17)	C6—C7	1.4828 (18)
N4—C7	1.3800 (19)	C10—H10A	0.9600
N4—H4	0.85 (2)	C10—H10B	0.9600
C1—C2	1.3942 (17)	C10—H10C	0.9600
			
C9—S1—C10	100.67 (7)	C4—C5—C6	120.60 (13)
C8—N1—N2	109.76 (10)	C4—C5—H5	119.7
C8—N1—C1	123.49 (11)	C6—C5—H5	119.7
N2—N1—C1	126.72 (10)	C5—C6—C1	118.70 (12)
C9—N2—N1	101.13 (10)	C5—C6—C7	120.00 (12)
C8—N3—C9	102.03 (11)	C1—C6—C7	121.28 (13)
C8—N4—C7	123.00 (11)	O1—C7—N4	121.17 (13)
C8—N4—H4	114.7 (15)	O1—C7—C6	123.63 (14)
C7—N4—H4	122.3 (15)	N4—C7—C6	115.18 (12)
N1—C1—C2	122.47 (12)	N3—C8—N1	111.11 (11)
N1—C1—C6	116.55 (11)	N3—C8—N4	128.60 (11)
C2—C1—C6	120.97 (12)	N1—C8—N4	120.29 (12)
C3—C2—C1	118.83 (13)	N2—C9—N3	115.95 (12)
C3—C2—H2	120.6	N2—C9—S1	125.39 (9)
C1—C2—H2	120.6	N3—C9—S1	118.65 (10)
C2—C3—C4	121.01 (13)	S1—C10—H10A	109.5
C2—C3—H3	119.5	S1—C10—H10B	109.5
C4—C3—H3	119.5	H10A—C10—H10B	109.5
C5—C4—C3	119.85 (13)	S1—C10—H10C	109.5
C5—C4—H4A	120.1	H10A—C10—H10C	109.5
C3—C4—H4A	120.1	H10B—C10—H10C	109.5
			
C8—N1—N2—C9	0.85 (13)	C5—C6—C7—O1	−1.4 (2)
C1—N1—N2—C9	179.10 (12)	C1—C6—C7—O1	−179.72 (14)
C8—N1—C1—C2	−175.64 (12)	C5—C6—C7—N4	177.42 (13)
N2—N1—C1—C2	6.33 (19)	C1—C6—C7—N4	−0.88 (19)
C8—N1—C1—C6	3.12 (18)	C9—N3—C8—N1	1.23 (14)
N2—N1—C1—C6	−174.91 (11)	C9—N3—C8—N4	−178.64 (14)
N1—C1—C2—C3	179.92 (12)	N2—N1—C8—N3	−1.39 (15)
C6—C1—C2—C3	1.2 (2)	C1—N1—C8—N3	−179.71 (11)
C1—C2—C3—C4	0.7 (2)	N2—N1—C8—N4	178.50 (11)
C2—C3—C4—C5	−1.6 (2)	C1—N1—C8—N4	0.17 (19)
C3—C4—C5—C6	0.6 (2)	C7—N4—C8—N3	175.64 (14)
C4—C5—C6—C1	1.3 (2)	C7—N4—C8—N1	−4.2 (2)
C4—C5—C6—C7	−177.05 (13)	N1—N2—C9—N3	−0.07 (14)
N1—C1—C6—C5	179.03 (12)	N1—N2—C9—S1	179.08 (9)
C2—C1—C6—C5	−2.2 (2)	C8—N3—C9—N2	−0.71 (15)
N1—C1—C6—C7	−2.66 (18)	C8—N3—C9—S1	−179.93 (9)
C2—C1—C6—C7	176.13 (12)	C10—S1—C9—N2	2.50 (13)
C8—N4—C7—O1	−176.73 (14)	C10—S1—C9—N3	−178.36 (11)
C8—N4—C7—C6	4.4 (2)		

### Hydrogen-bond geometry (Å, º)

**Table:**

*D*—H···*A*	*D*—H	H···*A*	*D*···*A*	*D*—H···*A*
N4—H4···N3^i^	0.85 (2)	2.05 (2)	2.896 (2)	174 (2)

Symmetry code: (i) −*x*, −*y*+1, −*z*.
